# Body mass index and gestational weight gain: relevance in gestational diabetes and outcomes - A retrospective cohort study

**DOI:** 10.20945/2359-3997000000463

**Published:** 2022-04-19

**Authors:** Patrícia Mendonça Oliveira Rosinha, Rosa Alexandra Barbosa Dantas, Márcia Inês Paiva Alves, Teresa Cristina Maia Ferreira Azevedo, Isabel Maria Ramos Inácio, Sara Gabriela Esteves Ferreira, Carla Alexandra Vieira Pedrosa, Marília Sousa Ferreira, Isabel Maria Albuquerque Sousa, Joana Guimarães Martins da Costa

**Affiliations:** 1 Centro Hospitalar Baixo Vouga Departmento de Endocrinologia Aveiro Portugal Departmento de Endocrinologia, Centro Hospitalar Baixo Vouga, Aveiro, Portugal; 2 Centro Hospitalar Baixo Vouga Departmento de Nutrição Aveiro Portugal Departmento de Nutrição, Centro Hospitalar Baixo Vouga, Aveiro, Portugal

**Keywords:** Gestational diabetes, body mass index, gestational weight gain, maternal obesity, blood glucose levels

## Abstract

**Objective:**

To evaluate the influence of maternal pre-pregnancy body mass index (BMI) and gestational weight gain (GWG) on blood glucose levels at diagnosis of gestational diabetes mellitus (GDM) and obstetric/neonatal outcomes.

**Subjects and methods:**

Retrospective cohort study including 462 women with GDM and singleton pregnancy delivered in our institution between January 2015 and June 2018 and grouped according to BMI/GWG.

**Results:**

The diagnosis of GDM was more likely to be established in the 1^st^ trimester (T) in women with obesity than in normal-weight (55.8% vs 53.7%, p = 0.008). BMI positively and significantly correlated with fasting plasma glucose (FPG) levels in the 1^st^T (rs = 0.213, p = 0.001) and 2^nd^T (rs = 0.210, p = 0.001). Excessive GWG occurred in 44.9% women with overweight and in 40.2% with obesity (p < 0.001). From women with obesity, 65.1% required pharmacological treatment (p < 0.001). Gestational hypertension (GH) was more frequent in women with obesity (p = 0.016). During follow-up, 132 cesareans were performed, the majority in mothers with obesity (p = 0.008). Of the 17 large-for-gestational-age (LGA) birthweight delivered, respectively 6 and 9 were offsprings of women with overweight and obesity (p = 0.019). Maternal BMI had a predictive value only for macrosomia [aOR 1.177 (1.006-1.376), p = 0.041]. BMI and GWG positively correlated with birthweight (rs = 0.132, p = 0.005; rs = 0.188, p = 0.005).

**Conclusion:**

Maternal obesity is related with a major probability of diagnosis of GDM in 1^st^T, fasting hyperglycemia in 2^nd^T and a more frequent need for pharmacological therapy. Pre-gestational obesity is associated with GH, cesarean delivery and fetal macrosomia.

## INTRODUCTION

GDM is the most common endocrine disorder of pregnancy and its definition has changed over time. Since 2015, the American Diabetes Association (ADA) defined GDM as diabetes diagnosed in the second or third trimester of pregnancy that was not clearly overt diabetes prior to gestation based on the perception that early GDM frequently represents undiagnosed pre-pregnancy diabetes ( 1 – 4 ). Therefore, GDM definition, particularly in early pregnancy, remains a controversial issue. The World Health Organization (WHO) actually considers two categories of hyperglycaemia firstly recognized in pregnancy, diabetes mellitus (DM) and GDM, being the GDM glucose cut-off points lower ( 5 ).

GDM prevalence has been rising presumably due to the influence of factors such as the delay of childbearing (≥30 years) and obesity ( 2 ). Women with GDM are at increased risk of preeclampsia, cesarean delivery and future type 2 DM while their infants tend to have higher rates of macrosomia (birth weight >4,000g) and shoulder dystocia ( 6 , 7 ). Previous evidence also shows that women with an earlier diagnosis of GDM tend to have more risk factors for type 2 DM compared to those with later GDM, namely a higher pre-pregnancy BMI, and, this way, be associated with worse pregnancy outcomes ( 1 ).

Likewise, BMI and GWG are globally increasing, raising concern about its repercussions both in terms of maternal and neonatal outcomes ( 8 ). Previous studies evidence that women with overweight/obesity are at increased risk when compared with normal-weight and that maternal obesity (MO) is a major risk factor for pre-eclampsia, cesarean delivery, fetal macrosomia, preterm delivery, fetal death and neonatal intensive care unit (NICU) admission ( 9 ).

GDM and MO share metabolic characteristics such as increased insulin resistance, hyperglycemia and hyperinsulinemia, being both independently associated with adverse maternal and neonatal outcomes ( 10 ). The combination of GDM and MO seems to have a greater impact than either alone and the development of GDM is directly related to the increase of BMI ( 6 , 11 ).

Recommendations for GWG in pregnancy by the Institute of Medicine (IOM) were updated on 2009 and are adopted almost worldwide ( Table 1 ) ( 12 ). Excessive GWG tends to worsen hyperglycemia and is associated with an increased risk of GH, GDM, pharmacological treatment need, cesarean section, maternal weight retention after labor, LGA infants and NICU admissions. On the other hand, insufficient GWG tends to result in lower rates of macrosomia, higher rates of prematurity and small-for-gestational-age (SGA) infants, despite the limited number of studies ( 7 , 13 – 15 ).

**Table 1:** 2009 Institute of Medicine (IOM) Recommendations for gestational weight gain during pregnancy

Pre-pregnancy BMI (kg/m^2^)	Recommended GWG (kg)
Underweight (BMI < 18.5 kg/m^2^)	12.5-18.0
Normal weight (18.5 ≤ BMI < 25 kg/m^2^)	11.5-16.0
Overweight (25 ≤ BMI < 30 kg/m^2^)	7.0-11.5
Obese (BMI ≥ 30 kg/m^2^)	5.0-9.0

Footnote: BMI: body mass index; GWG: gestational weight gain; kg: kilograms; kg/m^2^: kilograms per square meter.

In this context, it is crucial to understand which, between pre-pregnancy BMI and GWG, most influences maternal and neonatal outcomes. Therefore, the purpose of this study is to evaluate and compare the influence of maternal pre-pregnancy BMI and GWG on blood glucose levels in women diagnosed with GDM as well as maternal and neonatal outcomes.

## SUBJECTS AND METHODS

Retrospective cohort study including women with GDM and follow-up at Centro Hospitalar Baixo Vouga between 1^st^ January 2015 and 30^th^ June 2018. The following information was collected anonymously from medical recordings: demographic characteristics, medical and obstetric history, information related to the course of pregnancy and perinatal outcomes. The study protocol was in conformance with the World Medical Association's Helsinki Declaration and was approved by the Hospital's Ethics Committee.

**Figure 1:**
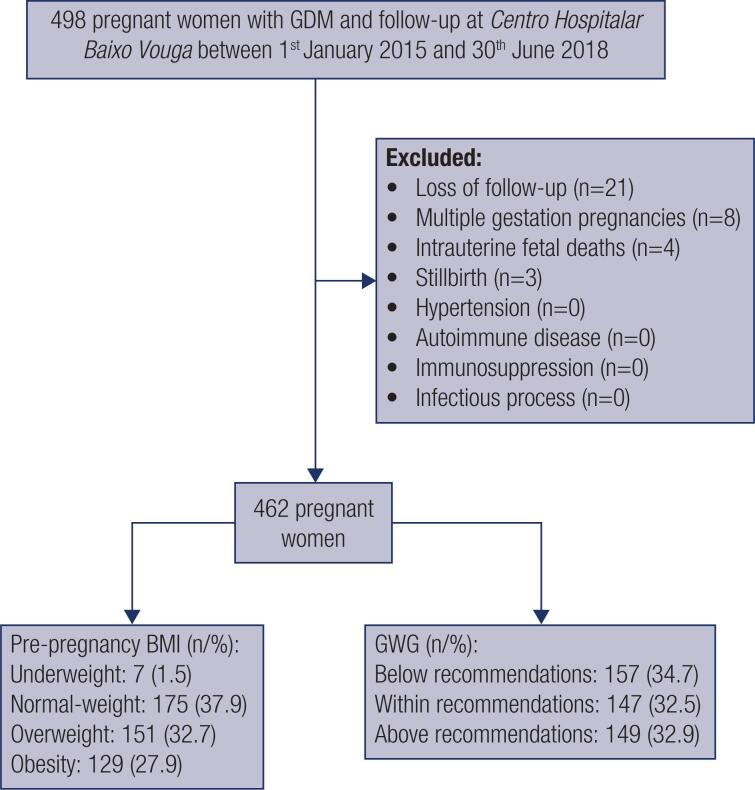
Study flow chart. Data presented as n (%). Footnote: BMI: body mass index; GDM: gestational diabetes mellitus; GWG: gestational weight gain.

The height was measured and the self-reported pre-pregnancy body weight of each pregnant women was recorded at the first antenatal visit and used to calculate the pre-pregnancy BMI [as weight (kg)/height (m)^2^]. GWG was calculated by subtracting each women's pre-pregnancy weight from her weight at delivery. The 462 eligible pregnant women were divided in categories according to pre-pregnancy BMI: underweight (<18,5 kg/m^2^), normal-weight (18,5–24,9 kg/m^2^), overweight (25–29,9 kg/m^2^) and obesity (≥30 kg/m^2^) and, posteriorly, attending to GWG relative to IOM Guidelines: below, within or above recommendations ( Table 1 ) ( 12 ). Exclusion criteria included: history of hypertension or autoimmune disease, users of immunosuppressive drugs (namely in a post-transplant setting), cases of loss of follow-up (in the absence of information regarding the course of the pregnancy or obstetric/neonatal outcomes if the delivery took place at another institution), multiple gestation pregnancies, infectious process at the time of screening, stillbirths and fetal deaths ( Figure 1 ).

Table 2 shows sample distribution according to BMI and GWG categories. A subgroup analysis was performed by dividing pregnant women in the following groups: 1 (BMI < 25 kg/m^2^ and normal GWG), 2 (BMI < 25 kg/m^2^ and excessive GWG), 3 (BMI ≥ 25 kg/m^2^ and normal GWG) and 4 (BMI ≥ 25 kg/m^2^ and excessive GWG). These subgroups were compared two by two, in the pairs: groups 1 vs 2, 3 vs 4, 1 vs 3 and 2 vs 4.

**Table 2:** Sample distribution according to BMI and GWG categories

	Underweight	Normal-weight	Overweight	Obesity	Total
GWG below recommendations	4 (0.9)	80 (17.7)	30 (6.6)	43 (9.5)	157 (34.7)
GWG within recommendations	3 (0.7)	60 (13.2)	51 (11.2)	33 (7.3)	147 (32.4)
GWG above recommendations	0	33 (7.3)	66 (14.6)	50 (11.0)	149 (32.9)
					453 (100.0)

Data presented as n (%).

Footnote: GWG: gestational weight gain.

Glucose level assessment was performed using the ADVIA® Chemistry Glucose Hexokinase_3 (GLUH_3) assay, with a sensitivity of 4 mg/dL (0.2 mmol/L), an adult reference range of 74-106 mg/dL and a coefficient of variation of less than 1%. GDM screening was established in the 1^st^T if FPG ≥ 5.1 mmol/L, or in the 2^nd^T by using a 75 g oral glucose tolerance test (OGTT) between 24-28 weeks of gestation with the diagnostic criteria proposed by the International Association of Diabetes Pregnancy Study Group (IADPSG): FPG ≥ 5.1 mmol/L and/or 1-hour glucose value ≥ 10.0 mmol/L and/or 2-hour glucose value ≥ 8.5 mmol/L ( 1 ). Regarding treatment, it was considered pharmacological if it included glucose-lowering therapy either oral or injectable in addition to nutritional treatment, whereas non-pharmacological treatment referred exclusively to nutritional therapy.

The following obstetric and neonatal outcomes were analysed: pre-eclampsia, hydramnios, GH, cesarean delivery, LGA (defined as birth weight above 90^th^ percentile of mean weight corrected for fetal sex and gestational age), prematurity (<37 weeks of gestation), neonatal morbidity [including neonatal hypoglycemia, neonatal hyperbilirubinemia, neonatal respiratory distress syndrome (RDS) and/or NICU admissions].

Sample size calculation was performed separately for BMI and GWG analyses by using the OpenEpi version 3 calculator for cohort studies and considering α = 0.05 and β = 0.10. Respectively, a sample size of 333 and 346 women would be needed to provide 90% power for BMI and GWG analyses using the method of Kelsey and cols. Thus, in order to obtain a power of 90%, a smaller sample size would be necessary than what was available.

Data analysis was performed using the statistical package IBM SPSS Statistics, version 20.0.0 (IBM Corp., Armonk, NY, USA). Categorical variables are presented as frequencies and percentages and continuous variables as means and standard deviations (SD) or medians and interquartile ranges (IQR) for variables with skewed distributions. Normal distribution was checked using Shapiro-Wilk test or skewness and kurtosis as appropriate. All reported p-values are two-tailed, with a p < 0.05 indicating statistical significance. Categorical variables were compared with the Pearson's Qui-square test or Fisher's exact test as appropriate. Spearman's correlation coefficient (rs) was used to assess the correlation between pre-pregnancy BMI or GWG and other variables. The interpretation of correlations' strength was made using the reference values provided by Bryman and Cramer in 1995. Logistic regression was used to evaluate whether pre-gestational BMI had predictive value for each of the neonatal outcomes, by adjusting for possible confounders.

## RESULTS

Of the total pregnancies followed in our institution, 462 met the criteria for this study. Primiparae accounted for 36.6% of deliveries, mean maternal age was 32.66 ± 5.46 years and the median BMI was 26.67 (7.34) kg/m^2^. At time of conception, the total number of pregnant women by BMI category was 7 for underweight, 175 for normal-weight, 151 for overweight, and 129 for obesity ( Figure 1 ).

Table 3 shows maternal characteristics, GDM particularities and adverse obstetric/neonatal outcomes categorized by BMI. Almost all women included were of European ethnic origin (98.9%, p = 0.146). There were no significant differences between those groups in terms of previous history of macrosomia or GDM. In terms of GDM diagnosis, women with obesity were more prone to diagnosis in the 1^st^T than normal-weight (55.8% vs 53.7%, p = 0.008). Of the total pregnancies followed, 230 women (49.8%) were diagnosed with GDM in the 1^st^T, pharmacological therapy was required in 48.7% cases and 32.2% pregnant women acquired excessive GWG. Women with pre-pregnancy obesity required more often pharmacological treatment (65.1%, p < 0.001) and developed more frequently GH (10.1%, p = 0.016). The percentage of family history of diabetes in 1^st^ degree relatives was lower in normal-weight pregnant women when compared to all other categories (p = 0.010). Excessive GWG was significantly less frequent in women with pre-pregnancy BMI < 25 kg/m^2^ when compared with those with overweight and obesity (p < 0.001). Cesarean delivery occurred in 40.3% women with obesity (p = 0.008). LGA infants were born more commonly from women with pre-gestational obesity (52.9%, p = 0.019). There were no significant differences between categories in terms of pre-eclampsia, hydramnios, prematurity or neonatal morbidity ( Table 3 ).

**Table 3:** Maternal characteristics, GDM particularities and adverse obstetric/neonatal outcomes categorized by BMI

		Underweight (n = 7)	Normal-weight (n = 175)	Overweight (n = 151)	Obesity (n = 129)	p value
**Maternal Characteristics**						
Ethnic origin:						
	European	7 (100.0)	175 (100.0)	147 (97.4)	128 (99.2)	0.146 a
	African	0	0	2 (1.3)	0	
	South American	0	0	1 (0.7)	0	
	Oriental Asian	0	0	0	1 (0.7)	
	South Asian	0	0	0	1 (0.8)	
Abortion on previous gestation		1 (14.3)	35 (20.0)	50 (33.1)	31 (24.1)	0.289 a
Macrosomia on previous gestation		1 (14.3)	4 (2.3)	11 (7.3)	7 (5.4)	0.074 a
GDM on previous gestation		1 (14.3)	18 (10.3)	20 (13.2)	23 (17.8)	0.254 a
Family history of diabetes in 1^st^ degree relatives		4 (57.1)	81 (46.3)	98 (64.9)	85 (65.9)	0.010 a
**GDM Particularities**						
Diagnosis at 1^st^ trimester		1 (14.3)	94 (53.7)	62 (41.1)	73 (55.8)	0.008 a
Pharmacological treatment		1 (14.3)	68 (38.9)	72 (47.7)	84 (65.1)	<0.001 a
**Obstetric Outcomes**						
Excessive GWG		0	33 (19.1)	66 (44.9)	50 (40.2)	<0.001 a
Pre-eclampsia		0	5 (2.9)	4 (2.6)	7 (5.4)	0.555 a
Hydramnios		0	0	3 (2.0)	3 (2.3)	0.192 a
Gestational Hypertension		0	8 (4.6)	8 (5.3)	13 (10.1)	0.016 a
Cesarean delivery		1 (14.3)	39 (22.3)	41 (27.2)	51 (40.3)	0.008 a
**Neonatal Outcomes**						
LGA birthweight		0	2 (1.1)	6 (4.0)	9 (7.0)	0.019 a
Prematurity		1 (14.3)	20 (11.5)	11 (7.3)	7 (5.4)	0.175 a
Neonatal morbidity		1 (14.3)	29 (16.6)	37 (24.7)	23 (17.8)	0.285 a
Neonatal Hypoglycemia		0	5 (2.9)	7 (4.7)	2 (1.6)	0.484 a
Neonatal Hyperbilirubinemia		0	24 (13.8)	22 (14.7)	18 (14.0)	0.933 a
Neonatal RDS		0	8 (4.6)	6 (4.0)	2 (1.6)	0.477 a
NICU admissions		0	19 (10.9)	21 (14.0)	8 (6.3)	0.170 a

Data presented as n (%).

Footnote: GDM: gestational diabetes mellitus; GWG: gestational weight gain; LGA: large-for-gestational-age; NICU: neonatal intensive care unit; RDS: respiratory distress syndrome.

a Fisher's exact test.

In what concerns GWG, 157 women were classified as below, 147 within and 149 above IOM recommendations. There were no significant differences between GWG categories in terms of maternal characteristics, GDM particularities or adverse obstetric/neonatal outcomes ( Table 4 ).

**Table 4:** Maternal characteristics, GDM particularities and adverse obstetric/neonatal outcomes categorized by GWG according to IOM Guidelines.

		Below recommendations (n = 157)	Within recommendations (n = 147)	Above recommendations (n = 149)	p value
**Maternal Characteristics**					
Ethnic origin:					
	European	156 (99.4)	146 (99.3)	146 (98.0)	0.613 a
	African	1 (0.6)	0	1 (0.7)	
	South American	0	1 (0.7)	0	
	Oriental Asian	0	1 (0.7)	0	
	South Asian	0	0	1 (0.7)	
Abortion on previous gestation		38 (24.2)	34 (23.1)	44 (29.5)	0.400 *
Macrosomia on previous gestation		9 (5.7)	8 (5.4)	4 (2.7)	0.408 *
GDM on previous gestation		19 (12.1)	25 (17.0)	18 (12.1)	0.362 *
Family history of diabetes in 1^st^ degree relatives		83 (52.9)	89 (60.5)	92 (61.7)	0.230 *
Pre-pregnancy obesity		43 (27.4)	33 (22.4)	50 (33.6)	0.104 *
**GDM Particularities**					
Diagnosis at 1^st^ trimester		79 (50.3)	72 (49.0)	75 (50.3)	0.964 *
Pharmacological treatment		77 (49.0)	73 (49.7)	72 (48.3)	0.979 *
**Obstetric Outcomes**					
Pre-eclampsia		6 (3.8)	4 (2.7)	5 (3.4)	0.946 a
Hydramnios		0	4 (2.7)	2 (1.3)	0.070 a
Gestational hypertension		11 (7.0)	6 (4.1)	11 (7.4)	0.385 *
Cesarean delivery		39 (24.8)	42 (28.6)	51 (34.2)	0.195 *
**Neonatal Outcomes**					
LGA birthweight		1 (0.6)	4 (2.7)	10 (6.7)	NA
Prematurity		16 (10.2)	15 (10.2)	8 (5.4)	0.222 *
Neonatal morbidity		27 (17.3)	39 (26.5)	22 (14.9)	0.331 *
Neonatal hypoglycemia		6 (3.8)	5 (3.4)	3 (2.0)	0.665 a
Neonatal hyperbilirubinemia		18 (11.5)	29 (19.7)	17 (11.5)	0.062 *
Neonatal RDS		4 (2.6)	6 (4.1)	5 (3.4)	0.718 a
NICU admissions		15 (9.7)	19 (12.9)	12 (8.1)	0.379 *

Data presented as n (%).

Footnote: GDM: gestational diabetes mellitus; LGA: large-for-gestational-age; NA: non-applicable; NICU: neonatal intensive care unit; RDS: respiratory distress syndrome.

* Pearson's qui-square.

a Fisher's exact test.

Positive, statistically significant and very weak correlations were identified between BMI and fasting glucose level in the 1^st^T (rs = 0.213, p = 0.001) and 2^nd^T (rs = 0.210, p = 0.001), even though no correlation was found between BMI and OGTT's glucose level at 60 and 120 minutes (rs = −0.005, p = 0.943; rs = −0.003, p = 0.960).

By adjusting for maternal age on logistic regression, BMI had a predictive value only for macrosomia (aOR 1.177, 95% CI 1.006-1.376, p = 0.041). BMI and GWG correlated positively, significantly and very weakly with birthweight (rs = 0.132, p = 0.005; rs = 0.188, p = 0.005 respectively) but not with gestational age (rs = −0.010, p = 0.833; rs = 0.069, p = 0.014).

Table 5 shows the results of the subgroup analysis comparing respectively women with BMI < 25 kg/m^2^ and BMI ≥ 25 g/m^2^ divided into the categories of normal/excessive GWG. There were no significant differences between groups 1 and 2 neither in terms of maternal characteristics, GDM particularities nor obstetric/neonatal outcomes ( Table 5 ). There were higher rates of neonatal morbidity in group 3 compared to group 4 (28.6% vs 15.7%, p = 0.035), with no significant differences in the other evaluated parameters. When comparing the two groups with normal GWG (groups 1 and 3), overweight women had more cesarean deliveries (35.7% vs 19.0%, p = 0.042) ( Table 5 ).

**Table 5:** Subgroup analysis comparing maternal characteristics, GDM particularities and adverse obstetric/neonatal outcomes between groups

	Group 1: BMI < 25 kg/m^2^ Normal GWG (n = 63)	Group2: BMI < 25 kg/m^2^ Excessive GWG (n = 33)	Group3: BMI ≥ 25 kg/m^2^ Normal GWG (n = 84)	Group 4: BMI ≥ 25 kg/m^2^ Excessive GWG (n = 116)
**Maternal Characteristics**				
European ethnic origin	63 (100.0)	33 (100.0)	83 (98.8)	113 (97.4)
Abortion on previous gestation	14 (22.2)	7 (21.2)	20 (23.8)	37 (67.2)
Macrosomia on previous gestation	2 (3.2)	1 (3.0)	6 (7.1)	3 (2.6)
GDM on previous gestation	10 (15.9)	1 (3.0)	15 (17.1)	17 (14.7)
Family history of diabetes in 1^st^ degree relatives	33 (52.4)	14 (42.4)	56 (66.7)	78 (67.2)
**GDM Particularities**				
Diagnosis at 1^st^ trimester	34 (54.0)	20 (60.6)	38 (45.2)	55 (47.4)
Pharmacological treatment	27 (42.9)	12 (36.4)	46 (54.8)	60 (51.7)
**Obstetric Outcomes**				
Pre-eclampsia	2 (3.2)	1 (3.0)	2 (2.4)	4 (3.4)
Hydramnios	0	0	4 (4.8)	2 (1.7)
Gestational Hypertension	1 (1.6)	2 (6.1)	3 (3.6)	9 (7.8)
Cesarean delivery	12 (19.0)	9 (27.3)	30 (35.7)	42 (36.2)
**Neonatal Outcomes**				
LGA birthweight	2 (3.2)	0	2 (2.4)	10 (8.6)
Prematurity	7 (11.1)	3 (9.1)	8 (9.5)	5 (4.3)
Neonatal morbidity	15 (23.8)	4 (12.1)	24 (28.6)	18 (15.7)
Neonatal Hypoglycemia	3 (4.8)	0	2 (2.4)	3 (2.6)
Neonatal Hyperbilirubinemia	12 (19.0)	4 (12.1)	17 (20.2)	13 (11.3)
Neonatal RDS	3 (4.8)	2 (6.1)	3 (3.6)	3 (2.6)
NICU admissions	11 (17.5)	3 (9.1)	8 (9.5)	9 (7.8)

Data presented as n (%).

Footnote: BMI: body mass index; GDM: gestational diabetes mellitus; GWG: gestational weight gain; LGA: large-for-gestational-age; NA: not applicable; NICU: neonatal intensive care unit; RDS: respiratory distress syndrome.

* Pearson's qui-square.

a Fisher's exact test.

## DISCUSSION

Our present findings confirm and extend previous reports from different countries linking GDM, pre-pregnancy overweight/obesity and GWG with adverse pregnancy outcomes ( 14 – 16 ). MO was associated with a higher probability of GDM diagnosis in the first and fasting hyperglycemia in the second trimester, a consequence of associated insulin resistance, and a heavier family history of diabetes in first-degree relatives. Women with obesity required more frequently pharmacological therapy and, similarly to previous studies, were associated with GH and cesarean deliveries ( 6 ), although there was no increase in the number of cases of pre-eclampsia or hydramnios in the obese category. Infants of women with obesity were, more frequently, LGA, even though there was no association with an increase of neonatal morbidity.

Previous studies proved fasting glucose to be the most accurate predictor of GDM as well as its increase in significance along with the pre-pregnancy BMI ( 17 ). Our results shown that this applies not only to fasting glucose at 1^st^T but also to the OGTT's glucose level at 0 minutes. Despite the low strength of these correlations, our results are in line with previous evidence and also demonstrate the association between BMI and birthweight, which is in agreement with the results of the HAPO study that shown a significant independent association of higher maternal glucose concentrations and MO with adverse pregnancy outcomes ( 11 ).

The results of the subgroup analysis reinforce the association between MO and the need for cesarean deliveries. Surprisingly, we also found a significantly higher neonatal morbidity in the group of infants born from mothers with a pre-pregnancy BMI ≥ 25 kg/m^2^ who had normal GWG compared to those with excessive GWG. Although current evidence on the impact of excessive GWG on obstetric and neonatal outcomes is sparse and controversial, it points towards its association with adverse obstetric and neonatal outcomes. In fact, this result cannot be explained with certainty in light of the current evidence and adds to the existing controversy. Nonetheless, a possible explanation might be the fact that current IOM recommendations for GWG are imperfect as they do not take into account ethnic groups or the existence of a diagnosis of GDM, which would have been important in a multiethnic population with GDM.

However, some limitations of our study should be noted. Firstly, its retrospective nature and the fact that cases of fetal death and stillbirth were not considered. The pre-pregnancy weight considered to calculate pre-pregnancy BMI was self-reported, which might have introduced a classification bias. Moreover, the sample size included might still have been insufficient to show an increase in neonatal morbidity.

In this study, BMI had a greater influence on both obstetric and neonatal outcomes compared to GWG. MO is an independent risk factor for adverse maternal and neonatal outcomes and, to a lesser degree, excessive GWG, as previously evidenced ( 18 , 19 ). There are different points of view concerning this as some other studies proved a greater impact for GWG ( 20 ). Yet, in addition to the growing evidence in favor of pre-pregnancy BMI compared to GWG, there are studies that inclusively suggest a greater utility of BMI unit change during pregnancy as a means to assess weight gain, especially in multiethnic populations, since it considers the height in its calculation ( 21 ). Still, we would say that these findings highlight the importance of having a healthy BMI at the time of conception and acquiring an appropriate GWG in order to prevent adverse pregnancy outcomes ( 10 , 22 ).

Further research is needed as opinions still diverge on this topic. However, we expect that these findings might be a step forward, reinforcing the association of pre-pregnancy overweight with adverse maternal and neonatal outcomes and placing it as the most important issue to be considered and prevented.

## References

[1] Cosson E, Carbillon L, Valensi P (2017). High Fasting Plasma Glucose during Early Pregnancy: A Review about Early Gestational Diabetes Mellitus. J Diabetes Res.

[2] Cruz-Hernández J, Hernández-García P, Lang-Prieto J, Yanes-Quesada M, Iglesias-Marichal I, Márquez-Guillén A (2016). Controversies in Screening and Diagnosis of Gestational Diabetes: Cuba's Position. MEDICC Rev.

[3] Mirghani Dirar A, Doupis J (2017). Gestational diabetes from A to Z. World J Diabetes.

[4] Association AD (2021). 2. Classification and Diagnosis of Diabetes: Standards of Medical Care in Diabetes – 2021. Diabetes Care.

[5] Alberti KG, Zimmet PZ (1998). Definition, diagnosis and classification of diabetes mellitus and its complications. Part 1: diagnosis and classification of diabetes mellitus provisional report of a WHO consultation. Diabet Med.

[6] Ray JG, Vermeulen MJ, Shapiro JL, Kenshole AB (2001). Maternal and neonatal outcomes in pregestational and gestational diabetes mellitus, and the influence of maternal obesity and weight gain: the DEPOSIT study. Diabetes Endocrine Pregnancy Outcome Study in Toronto. QJM.

[7] Viecceli C, Remonti LR, Hirakata VN, Mastella LS, Gnielka V, Oppermann MLR (2017). Weight gain adequacy and pregnancy outcomes in gestational diabetes: a meta-analysis. Obes Rev.

[8] Goldstein RF, Abell SK, Ranasinha S, Misso M, Boyle JA, Black MH (2017). Association of Gestational Weight Gain With Maternal and Infant Outcomes. JAMA.

[9] Hung TH, Hsieh TT (2016). Pregestational body mass index, gestational weight gain, and risks for adverse pregnancy outcomes among Taiwanese women: A retrospective cohort study. Taiwan J Obstet Gynecol.

[10] Cosson E, Cussac-Pillegand C, Benbara A, Pharisien I, Nguyen MT, Chiheb S (2016). Pregnancy adverse outcomes related to pregravid body mass index and gestational weight gain, according to the presence or not of gestational diabetes mellitus: A retrospective observational study. Diabetes Metab.

[11] HAPO Study Cooperative Research Group (2002). The Hyperglycemia and Adverse Pregnancy Outcome (HAPO) Study. Int J Gynaecol Obstet.

[12] (2009). Weight Gain During Pregnancy [Internet].

[13] Papazian T, Abi Tayeh G, Sibai D, Hout H, Melki I, Rabbaa Khabbaz L. (2017). Impact of maternal body mass index and gestational weight gain on neonatal outcomes among healthy Middle-Eastern females. PLoS One.

[14] Goldstein RF, Abell SK, Ranasinha S, Misso ML, Boyle JA, Harrison CL (2018). Gestational weight gain across continents and ethnicity: systematic review and meta-analysis of maternal and infant outcomes in more than one million women. BMC Med.

[15] Soltani H, Lipoeto NI, Fair FJ, Kilner K, Yusrawati Y (2017). Pre-pregnancy body mass index and gestational weight gain and their effects on pregnancy and birth outcomes: a cohort study in West Sumatra, Indonesia. BMC Womens Health.

[16] Xiao L, Ding G, Vinturache A, Xu J, Ding Y, Guo J (2017). Associations of maternal pre-pregnancy body mass index and gestational weight gain with birth outcomes in Shanghai, China. Sci Rep.

[17] Wang C, Zhu W, Wei Y, Su R, Feng H, Lin L (2016). The Predictive Effects of Early Pregnancy Lipid Profiles and Fasting Glucose on the Risk of Gestational Diabetes Mellitus Stratified by Body Mass Index. J Diabetes Res.

[18] Alberico S, Montico M, Barresi V, Monasta L, Businelli C, Soini V (2014). The role of gestational diabetes, pre-pregnancy body mass index and gestational weight gain on the risk of newborn macrosomia: results from a prospective multicentre study. BMC Pregnancy Childbirth.

[19] Dzakpasu S, Fahey J, Kirby RS, Tough SC, Chalmers B, Heaman MI (2015). Contribution of prepregnancy body mass index and gestational weight gain to adverse neonatal outcomes: population attributable fractions for Canada. BMC Pregnancy Childbirth.

[20] Chiou YL, Hung CH, Liao HY (2018). The Impact of Prepregnancy Body Mass Index and Gestational Weight Gain on Perinatal Outcomes for Women With Gestational Diabetes Mellitus. Worldviews Evid Based Nurs.

[21] Padmanabhan S, Wagstaff A, Tung V, Chan YF, Bartlett A, Lau SM (2014). Increase in body mass index during pregnancy and risk of gestational diabetes. Diabetes Res Clin Pract.

[22] Kim SY, Sharma AJ, Sappenfield W, Wilson HG, Salihu HM (2014). Association of Maternal Body Mass Index, Excessive Weight Gain, and Gestational Diabetes Mellitus With Large-for-Gestational-Age Births. Obstet Gynecol.

